# Rumen and colon-centered microbiota-liver interactions associated with bile acid-related metabolic regulation in sheep

**DOI:** 10.7717/peerj.21705

**Published:** 2026-09-10

**Authors:** Yuyang Gao, Bing Wang

**Affiliations:** 1 State Key Laboratory of Animal Nutrition and Feeding, College of Animal Science and Technology, China Agricultural University, Beijing, China; 2 Sanya Institute, China Agricultural University, Sanya, Hainan, China

**Keywords:** Bile acid, Sheep, Rumen, Microbiota, Gut–liver axis, Metabolic regulation

## Abstract

**Background:**

Ruminant gastrointestinal microbiota are spatially heterogeneous, yet whether the rumen microbiota contributes to bile acid-related gut–liver associations beyond its local fermentative role remains unclear. Here, paired rumen, ileum, and colon microbiota were integrated with liver transcriptomic data, blood biochemical and tail fat-related phenotypes to determine which gastrointestinal sector microbiota play prioritized host interactions in a sheep model.

**Results:**

The rumen, ileum, and colon showed marked taxonomic and predicted functional divergence, indicating strong spatial restructuring along the digestive tract. Distinct patterns included rumen-enriched taxa, coordinated rumen–colon taxa, and taxa showing progressive shifts across compartments. Representative genera included *Prevotella_7* and *Butyrivibrio* in rumen-enriched patterns, *Anaerovibrio* and *Ruminococcus* in coordinated rumen–colon patterns, and *Fournierella* and *Treponema* in colon-enriched patterns, whereas *Olsenella* and *[Ruminococcus]_gauvreauii_group* showed cross-compartment continuity. Pairwise correlation analyses revealed compartment-specific microbiota–host association profiles: both the rumen and colon formed major interfaces with hepatic bile acid-related genes, whereas the rumen showed broader associations with blood metabolic traits and tail fat deposition. The structure associated with serum total bile acids was most prominently associated with hepatic scavenger receptor class B member 1 (*SCARB1*), whereas the structures associated with serum triglycerides and tail fat mass were most prominently associated with hepatic phosphoenolpyruvate carboxykinase 1 (*PCK1*) and hydroxyacyl-CoA dehydrogenase trifunctional multienzyme complex subunit beta (*HADHB*), respectively. Mediation analysis further provided statistical support for candidate intermediate associations involving hepatic genes between microbial variation and host phenotypes, with the strongest signals centered on serum total bile acids.

**Conclusions:**

These findings indicate that the rumen is not merely a local fermentation chamber but may represent an important upstream microbial component associated with the bile acid-related gut–liver axis. Our results support consideration of an expanded, rumen-inclusive framework for investigating bile acid-related gut–liver associations in ruminants.

## Introduction

Ruminant gastrointestinal ecosystems are spatially heterogeneous, with distinct microbial communities and metabolic activities distributed across different anatomical compartments (Cheng et al., 2022). Among these, the rumen is the major foregut fermentation chamber and contains a dense, metabolically active microbial community specialized in the degradation and transformation of complex plant substrates (Mizrahi, Wallace & Moraïs, 2021; Wang & Luo, 2021). Recent evidence further indicates that lignocellulose degradation in the rumen is mediated by coordinated microbial degradation cascades, with key degradation phases and associated microbial populations being strongly influenced by diet (Lin et al., 2024). By contrast, the small intestine is more closely linked to nutrient absorption, host transport processes, and host–microbe signaling (Ghosh et al., 2021). The hindgut also makes an important contribution to host metabolism. For example, the goat caecum harbours a distinct microbial community enriched in reductive acetogenesis, supporting a role in energy harvest (Li et al., 2025). Together, these observations indicate that microbial organization along the ruminant digestive tract is strongly compartmentalized, and that different gut regions are likely to differ not only in taxonomic composition but also in ecological function and relevance to host physiology.

In humans and monogastric animals, the gut–liver axis is widely recognized as a central framework linking intestinal microbial activity with hepatic metabolic regulation (Ringseis, Gessner & Eder, 2020). Within this framework, bile acids are of particular interest because they are not only essential for lipid digestion and absorption, but also act as signaling molecules that regulate energy metabolism, immune function, and host–microbiota interactions (Mohanty et al., 2024; Wahlström et al., 2016). Gut microbiota convert primary bile acids into secondary bile acids through microbial biotransformation. These microbiota-derived bile acids function as signaling molecules by activating intestinal and hepatic bile acid receptors, thereby regulating host metabolic homeostasis. Most bile acids are subsequently reabsorbed into the portal circulation and returned to the liver through enterohepatic circulation (Larabi, Masson & Bäumler, 2023; Mori et al., 2022). Consequently, bile acid metabolism and signaling constitute a central mechanism of gut–liver communication. Most previous studies have focused on the small intestine, especially the ileum, because this region plays a major role in bile acid reabsorption and feedback control of hepatic bile acid synthesis (Wang et al., 2024). The large intestine has also been implicated in microbial bile acid metabolism through its dense and metabolically versatile microbiota (Chinda et al., 2022; Ridlon & Gaskins, 2024). This intestine-centered framework has substantially advanced current understanding of gut–liver communication. However, despite increasing recognition of microbiota–bile acid interactions, the coordinated relationships among gastrointestinal microbiota, hepatic bile acid metabolism-related transcriptional regulation, and host metabolic phenotypes remain poorly understood in ruminants.

Unlike monogastric species, ruminants possess a highly specialized foregut microbial organ that exerts profound effects on host metabolism. The rumen is not merely a local site of feed fermentation, but a functionally important microbial ecosystem with exceptional taxonomic diversity and metabolic capacity (Zhang et al., 2025). Its fermentation products, particularly short-chain fatty acids, make major contributions to hepatic gluconeogenesis and *de novo* fatty acid synthesis in the host (Buitenhuis et al., 2019; Li et al., 2023; Pang et al., 2023). In addition, recent evidence suggests that the rumen microbiome can transform bile acids and thereby influence systemic metabolic regulation (Zhang et al., 2024a). These findings suggest that the ruminant gut–liver axis may include an upstream ruminal microbial input in addition to downstream intestinal contributions.

However, which gastrointestinal compartment shows the strongest associations with hepatic bile acid-related features in sheep remains poorly defined. It is unclear whether the rumen primarily acts as an isolated upstream fermentation compartment, whether it contributes to downstream microbial organization, or whether these gastrointestinal segments jointly form a coordinated microbial network linked to liver function. A comparative and integrated analysis across these compartments is therefore needed to refine the current framework of gut–liver interactions in ruminants. Here, we used paired ruminal, ileal, and colonic digesta samples from the same sheep and integrated microbial data with hepatic bile acid-related gene expression, blood biochemical traits, and tail fat phenotypes within a unified multi-omics framework. We first characterized taxonomic and predicted functional divergence across the three compartments and identified genera showing coordinated cross-compartment variation. We then examined associations of these microbial patterns with liver transcriptional profiles and host metabolic phenotypes to characterize potential compartment-specific microbiota–host association structures. Through correlation analysis, sparse generalized canonical correlation analysis (sGCCA), and mediation analysis, we sought to evaluate whether bile acid-related metabolic variation in sheep shows stronger statistical association with rumen-centered, intestine-centered, or coordinated multi-compartment gut–liver patterns.

## Materials and Methods

### Experimental design and data collection

All animal procedures were approved by the Animal Care Committee of China Agricultural University (Beijing, China; approval no. AW30901202-1-1). A total of 30 male Tan lambs (*Ovis aries*; approximately 9 months old; body weight 36.4 ± 3.54 kg, mean ± SD) were included in this study. All animals were obtained from Ningxia Tianyuan Sheep Farm, Hongsibu District, Ningxia Hui Autonomous Region, China, maintained under the same experimental conditions, housed in group pens at a density of five lambs per pen, and managed under routine farm conditions. The lambs were fed two different basal diets formulated as a total mixed ration. The complete ingredient composition and nutrient levels of the basal diet are presented in Table S2. The lambs were fed twice daily at 09:00 and 17:00 and had *ad libitum* access to feed and clean drinking water. No additional environmental enrichment beyond routine group housing and standard farm management was applied.

No surgical procedure, disease-inducing treatment, or other painful experimental intervention was performed before slaughter; therefore, analgesia was not administered. At the end of the experiment, the lambs were slaughtered according to Muslim halal slaughter procedures, as specified in the approved animal ethics protocol. Blood was collected from the jugular vein before slaughter, and rumen, ileal, and colonic contents, liver tissue, and tail fat samples were collected immediately after slaughter, frozen in liquid nitrogen, and stored at −80 °C until analysis. Animals were monitored under routine farm management throughout the experiment, and no unexpected adverse events were recorded. Because no disease-inducing procedure or survival surgery was performed, no procedure-specific humane endpoint was required before scheduled slaughter. No separate treatment or control group was established because this study was designed as an observational, paired multi-omics analysis of gastrointestinal compartments within the same animals rather than as a treatment-controlled intervention experiment. The individual animal was considered the experimental unit. The sample size was determined by the availability of animals with complete matched ruminal, ileal, colonic, liver, blood biochemical, and tail fat phenotype data from the shared experimental cohort. Animals were included when complete paired gastrointestinal samples and corresponding host phenotypic data were available. No animals were excluded before analysis; individual features or samples were retained or removed only according to the data-processing criteria described below. Because no treatment allocation was performed, randomization to experimental groups was not applicable. Samples from the same animal were treated as matched observations to reduce between-animal confounding. Blinding was not specifically applied during sample collection or bioinformatic analysis.

Microbiota from the rumen, ileum, and colon of the same animal were treated as matched samples for within-animal comparisons. Host phenotypic data included five blood biochemical traits and three tail fat-related traits. The blood biochemical trait matrix comprised total cholesterol (TC), triglycerides (TG), low-density lipoprotein cholesterol (LDL), total bile acids (TBA), and glucose (GLU). Tail fat-related phenotypes included tail fat mass (TailFat), tail fat per carcass weight (TailFat/Carcass), and tail fat per live weight (TailFat/LiveWeight). Blood biochemical traits and tail fat phenotypes were obtained from previous studies (Yu et al., 2026; Zhang et al., 2022). Blood was collected from the jugular vein 6 h after the morning feeding and centrifuged at 3,000× *g* for 10 min to separate serum. Serum TC, TG, LDL, TBA, and GLU were measured using commercial kits from Nanjing Jiancheng Bioengineering Institute (Nanjing, China) according to the manufacturer’s instructions. Serum TBA represented the aggregate concentration of circulating bile acids. Tail fat and carcass weights were recorded immediately after slaughter, and tail fat-related ratios were calculated relative to carcass weight or live weight. Raw liver transcriptome sequencing reads are available in the NCBI Sequence Read Archive (SRA) under accession number PRJNA1158691. Based on Kyoto Encyclopedia of Genes and Genomes (KEGG) pathway annotations, hepatic genes involved in bile acid signaling and metabolism and related glycolipid metabolism pathways were identified and extracted from the liver transcriptome dataset for downstream analyses focusing on bile acid-related metabolic associations. The relative mRNA expression levels of the selected genes are provided in Table S1. These expression values were derived from the RNA-sequencing dataset. This was an exploratory multi-omics association study; no single primary outcome measure was specified *a priori*. Microbial community structure, predicted microbial function, hepatic bile acid-related gene expression, blood biochemical traits, and tail fat phenotypes were jointly assessed as outcome-related variables.

### Paired analysis of microbial structure and function from three gastrointestinal compartments

Microbial DNA was extracted from ruminal, ileal, and colonic contents using the HiPure Stool DNA Kit (Magen, Guangzhou, China), according to the manufacturer’s instructions. The V3–V4 hypervariable region of the bacterial 16S rRNA gene was amplified using the forward primer (5′-ACTCCTACGGGAGGCAGCA-3′) and the reverse primer (5′-GGACTACHVGGGTWTCTAAT-3′). The procedures for 16S rRNA gene sequencing and raw data annotation were the same as those used in the shared sample cohort described previously (Zhao et al., 2025). The 16S rRNA sequencing data generated from rumen, ileum, and colon samples in this study were deposited in the NCBI Sequence Read Archive under accession numbers PRJNA1156902, PRJNA1156904, and PRJNA1158020, respectively.

Alpha-diversity analyses were conducted using matched rumen–ileum–colon samples from the same animals. Microbial richness and diversity were assessed using observed features, ACE, Chao1, Simpson, Shannon, and phylogenetic diversity indices after retaining complete paired sets across the three compartments. Overall differences among compartments were tested using the Friedman test, with Benjamini–Hochberg correction applied across indices. Beta diversity was calculated using binary Jaccard and Bray–Curtis distances and visualized by principal coordinates analysis (PCoA). Differences in community structure among gastrointestinal compartments were evaluated using permutational analysis of variance (PERMANOVA), and homogeneity of multivariate dispersion was assessed using permutational analysis of multivariate dispersions (PERMDISP).

Genus-level differential abundance analysis was performed in a three-compartment framework using ANCOM-BC2 (Lin & Peddada, 2020). Intestinal site was modelled as a fixed effect and SheepID as a random effect to account for repeated sampling within animals. The analysis was run with prv_cut = 0.10, lib_cut = 1,000, and struc_zero = FALSE, and Holm correction was used to control for multiple testing. To further define spatial distribution patterns, genus-level abundance differences across compartments were also evaluated using the Friedman test, followed by BH-adjusted pairwise Wilcoxon signed-rank tests for *post hoc* comparisons. Genera with an overall adjusted *q* < 0.001 were considered significantly different among compartments, and pairwise comparisons with adjusted *q* < 0.001 were used for downstream pattern assignment. Median relative abundance across the rumen, ileum, and colon was used to classify compartmental distribution patterns.

Microbial functional profiles were inferred using PICRUSt2 (Douglas et al., 2020), and KEGG Orthology (KO) abundance tables were used for downstream analyses. These profiles represent predicted gene-family and pathway potential inferred from 16S rRNA-based taxonomic composition and reference genomes, rather than directly measured microbial genes, transcripts, proteins, or metabolic activities. KO features were filtered by prevalence and mean abundance before testing. Significantly retained KOs were subsequently summarized into pathway, level 2, and level 1 functional categories based on offline KEGG mapping files. Differential predicted functional potential analyses were then performed separately at the KO, pathway, level 2, and level 1 levels, with Benjamini–Hochberg correction used to control for multiple testing. Features with adjusted *q* < 0.001 were considered significant.

### Identification of coordinated genera across gastrointestinal compartments

To identify genera showing coordinated variation across the gastrointestinal tract, genus-level abundance tables from matched ruminal, ileal, and colonic samples were organized by SheepID. For each genus, prevalence, non-zero sample count, and mean abundance were calculated separately within each compartment. In the main analysis, genera were required to reach a prevalence threshold of at least 0.50 in one or more compartments to ensure sufficient representation. Cross-compartment coordination was assessed using Spearman correlation analysis across matched animals, with significance defined as BH-adjusted *q* < 0.05. For structural classification, |*r*| ≥ 0.30 was considered strong evidence of coordinated variation, whereas |*r*| ≥ 0.20 was considered supportive evidence in the third compartment when defining continuity patterns. This strategy enabled classification of genera into patterns reflecting continuity between adjacent compartments, broader multi-segment coordination, or localized compartment-specific behavior.

### Multi-omics correlation and integrative analysis

Before multi-omics integration, all data matrices were aligned using common sample identifiers. Blood biochemical traits and tail fat phenotypes were standardized by z-score transformation. Microbiome matrices from the rumen, ileum, and colon were processed separately using a unified workflow consisting of prevalence filtering at 0.50, count zero multiplicative replacement (CZM) (Martín-Fernández et al., 2015), centered log-ratio (CLR) transformation, and *z*-score standardization. Liver transcriptome data, based on the formal_log2TPM matrix, were standardized in the same way. Preliminary pairwise association analyses were then performed to define the three-layer framework linking microbiota, liver genes, and host phenotypes before multiblock integration. Eleven pairwise modules were examined: Rum *vs*. Liver, Ile *vs*. Liver, Col *vs*. Liver, Rum *vs*. Blood, Ile *vs*. Blood, Col *vs*. Blood, Rum *vs*. TailFat, Ile *vs*. TailFat, Col *vs*. TailFat, Liver *vs*. Blood, and Liver *vs*. TailFat. Spearman’s rank correlations were calculated for all feature pairs, and associations with BH-adjusted *q* < 0.05 and |ρ| ≥ 0.30 were retained as significant.

To identify coordinated cross-omics structures, sparse generalized canonical correlation analysis (sGCCA) was performed across five blocks: ruminal microbiota, ileal microbiota, colonic microbiota, liver transcriptome, and one host phenotype at a time. Separate unsupervised models were fitted for GLU, TC, TG, TBA, LDL, TailFat, TailFat/Carcass, and TailFat/LiveWeight. Within each model, microbiome blocks were filtered at a prevalence threshold of 0.50, processed by CZM zero replacement and CLR transformation, and standardized by z score. The liver transcriptome block and the phenotype block were standardized in the same manner. Only samples shared across all five blocks were retained. A fully connected design matrix was specified, and one latent component was extracted from each block.

For biological interpretation, all trait-specific sGCCA outputs were further processed through a structured workflow to evaluate model support, extract core features from each block, reconstruct source–liver–trait chains, and identify liver hub genes connected to multiple upstream microbial features.

### Mediation analysis

Mediation analysis was performed to evaluate statistical evidence for candidate intermediate associations involving liver molecular traits in microbiota–host relationships, using the R package mediation (Tingley et al., 2014). In these models, ruminal, ileal, or colonic genera were treated as source variables, liver genes as mediators, and eight host traits—TC, TG, LDL, TBA, GLU, TailFat, TailFat/Carcass, and TailFat/LiveWeight—as outcomes. Microbiome data were preprocessed as described for the pairwise analyses, including prevalence filtering at 0.50, CZM zero replacement, CLR transformation, and z-score standardization. Liver gene expression values from the formal_log2TPM matrix and host traits were also standardized to z scores before modelling. For each candidate source–mediator–outcome chain, linear models were fitted as mediator ~ source and trait ~ source + mediator, and mediation effects were estimated using nonparametric bootstrapping with 1,000 simulations. Because the data were observational and cross-sectional, the resulting mediation estimates were interpreted as statistical evidence for candidate intermediate relationships rather than as proof of causal mediation.

## Results

### Distinct microbial profiles and predicted functional divergence across the rumen, ileum, and colon

**Figure 1:**
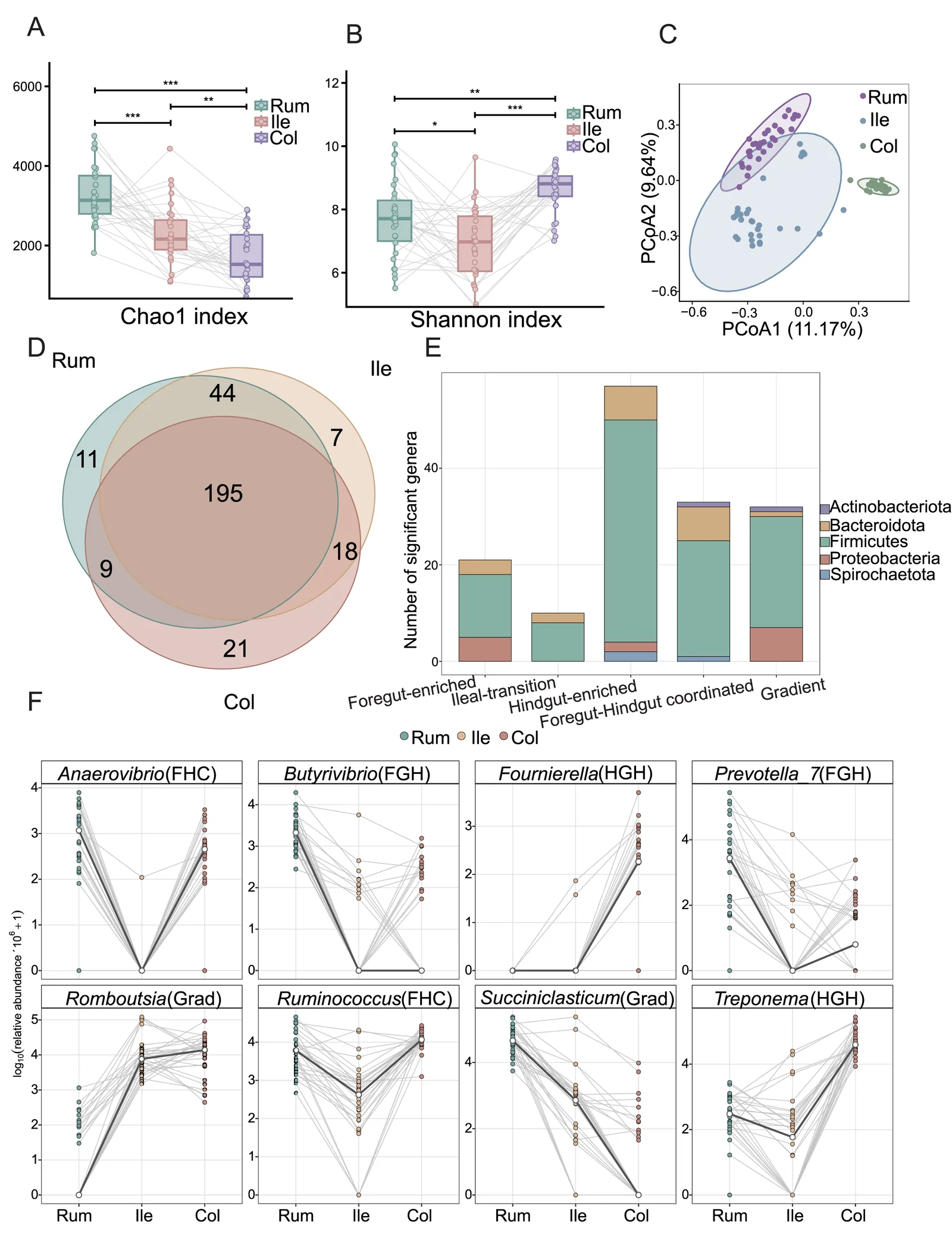
Microbial diversity and spatial taxonomic divergence across the rumen, ileum, and colon. (A) Chao1 index. (B) Shannon index. (C) Principal coordinates analysis based on Bray–Curtis distances. (D) Venn diagram showing the presence distribution of significant genera among the rumen, ileum, and colon. (E) Numbers of significant genera assigned to five spatial pattern classes. (F) Paired abundances of representative genera across the three compartments, including foregut-high (FGH), foregut–hindgut coordinated (FHC), gradient-pattern (Grad), hindgut-high (HGH), and ileal-transition genera (IT). Asterisks indicate statistical significance: *q < 0.05, **q < 0.01, and ***q < 0.001.

To characterize microbial communities across the digestive tract, we analyzed the V3–V4 region of the 16S rRNA gene in matched ruminal, ileal, and colonic samples. Alpha diversity differed markedly among the three compartments. The Chao1 index was highest in the rumen and lowest in the colon, whereas the Shannon index was highest in the colon and lowest in the ileum (Figs. 1A and 1B). These patterns indicate that the rumen harbored greater microbial richness, while the colon showed greater community evenness and overall diversity. Bray–Curtis-based principal coordinates analysis (PCoA) further demonstrated clear separation among the three compartments, with colon samples forming the tightest cluster (Fig. 1C). PERMANOVA confirmed a significant compartment effect (R^2^ = 0.321, F = 20.604, *P* = 0.0001), and PERMDISP indicated significant differences in within-group dispersion (F = 39.666, *P* = 0.0001). All pairwise PERMANOVA comparisons were significant, with the greatest separation observed between the rumen and colon. At the phylum level, Firmicutes and Bacteroidota dominated the microbial communities in the rumen, ileum, and colon. At the genus level, the rumen was mainly characterized by *Lysinibacillus*, *Escherichia_Shigella*, and *Acinetobacter*, the ileum by *Lysinibacillus*, *Bacillus*, and *Anaerocolumna*, and the colon by *Rikenellaceae_RC9_gut_group*, *UCG_005*, and *Treponema*.

A total of 305 genera showed significant overall differences across the rumen, ileum, and colon (Fig. 1D). Among these, 195 were detected in all three compartments, whereas a smaller number were restricted to a single compartment or shared between only two compartments, including 11 rumen-specific genera, seven ileum-specific genera, 21 colon-specific genera, 44 shared between the rumen and ileum, nine shared between the rumen and colon, and 18 shared between the ileum and colon (Fig. 1D). When these genera were classified according to their spatial distribution patterns, the colon-enriched pattern was the most common, comprising 60 genera, followed by 25 rumen-enriched genera, 34 rumen–colon coordinated genera, 34 gradient-change genera, and 10 ileum-transition genera (Fig. 1E). The trajectories of representative genera further illustrated this structured organization along the digestive tract (Fig. 1F). The rumen-enriched representative genera *Butyrivibrio* and *Prevotella_7* showed relatively high abundance in the rumen and then gradually declined toward the distal gut. The rumen–colon coordinated representative genera *Anaerovibrio* and *Ruminococcus* maintained relatively high abundance in both the rumen and colon but were markedly reduced in the ileum. The colon-enriched representative genera *Fournierella* and *Treponema* showed a progressive increase from the rumen to the colon, where their abundance reached the highest levels. Meanwhile, *Romboutsia* and *Succiniclasticum* displayed continuous gradient-like changes.

**Figure 2:**
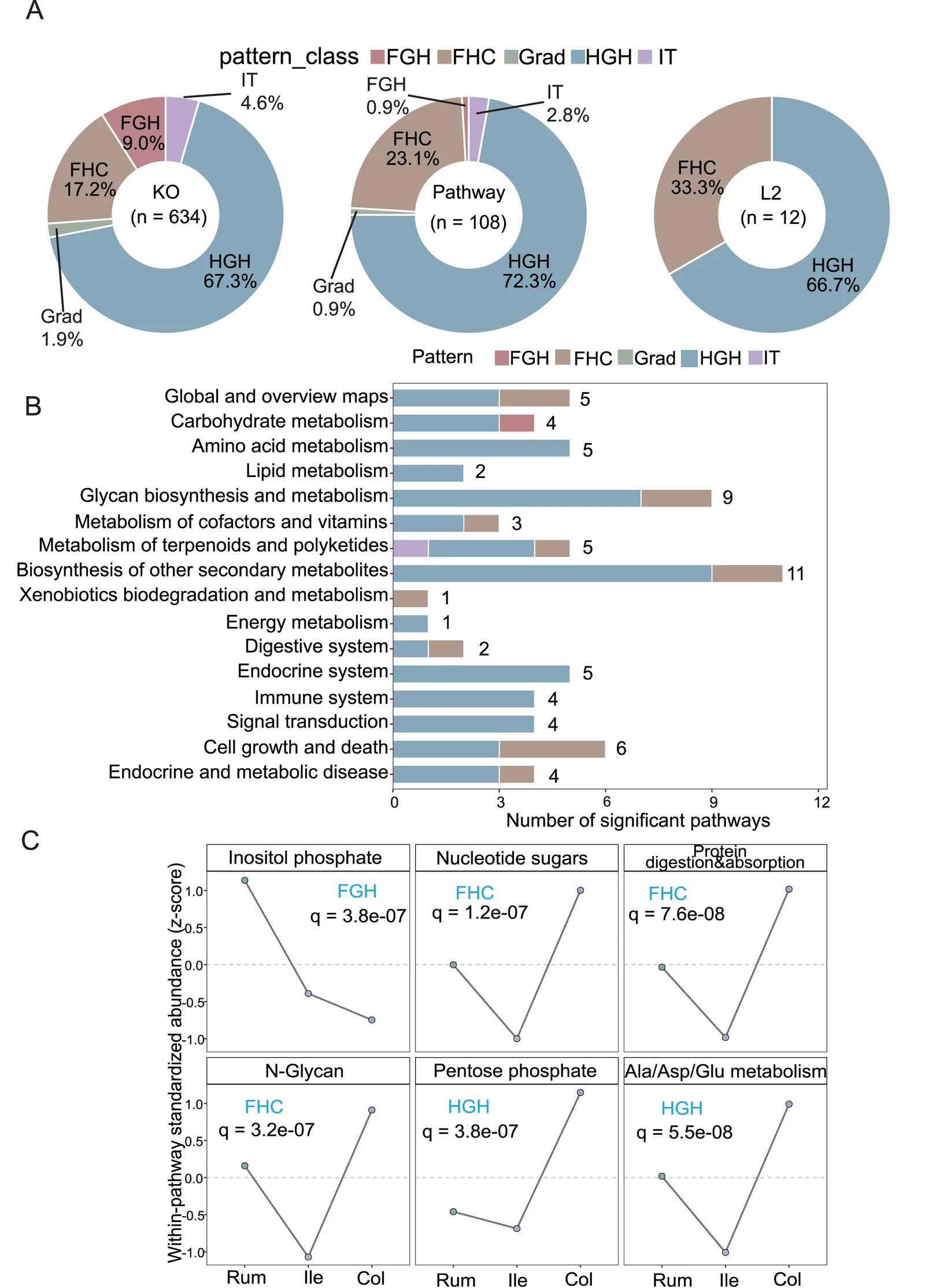
Predicted functional divergence across the rumen, ileum, and colon microbiota. (A) Distribution of differential features across spatial pattern classes at the KO, pathway, and KEGG level 2 category levels. (B) Numbers of significant pathways within major KEGG level 2 categories, stratified by spatial pattern class. (C) Standardized abundance trajectories of representative PICRUSt2-predicted pathways across the three compartments.

Significant differences were detected in the PICRUSt2-predicted profiles at the ortholog, pathway, and Level 2 functional category levels, with 634 orthologs, 108 pathways, and 12 Level 2 categories identified, respectively. The colon-enriched pattern again predominated, accounting for 67.4%, 72.2%, and 66.7% of significant features at these three levels, respectively. The rumen–colon coordinated pattern ranked second, accounting for 23.1% of pathways and 33.3% of Level 2 categories, whereas the rumen-enriched pattern represented only a small fraction (Fig. 2A). Classification of the significant predicted pathways showed enrichment in broad metabolic categories, including global and overview maps, carbohydrate metabolism, amino acid metabolism, lipid metabolism, glycan biosynthesis and metabolism, metabolism of cofactors and vitamins, biosynthesis of other secondary metabolites, signal transduction, and immune- and endocrine-related pathways (Fig. 2B). The predicted potential for inositol phosphate metabolism was relatively higher in the rumen and then declined distally. The rumen–colon coordinated pattern included the predicted pathways for biosynthesis of nucleotide sugars and protein digestion and absorption, whereas the colon-enriched pattern included the predicted pathways for the pentose phosphate pathway and alanine, aspartate, and glutamate metabolism (Fig. 2C).

### Cross-compartment continuity reveals an organized microbial continuum

**Figure 3:**
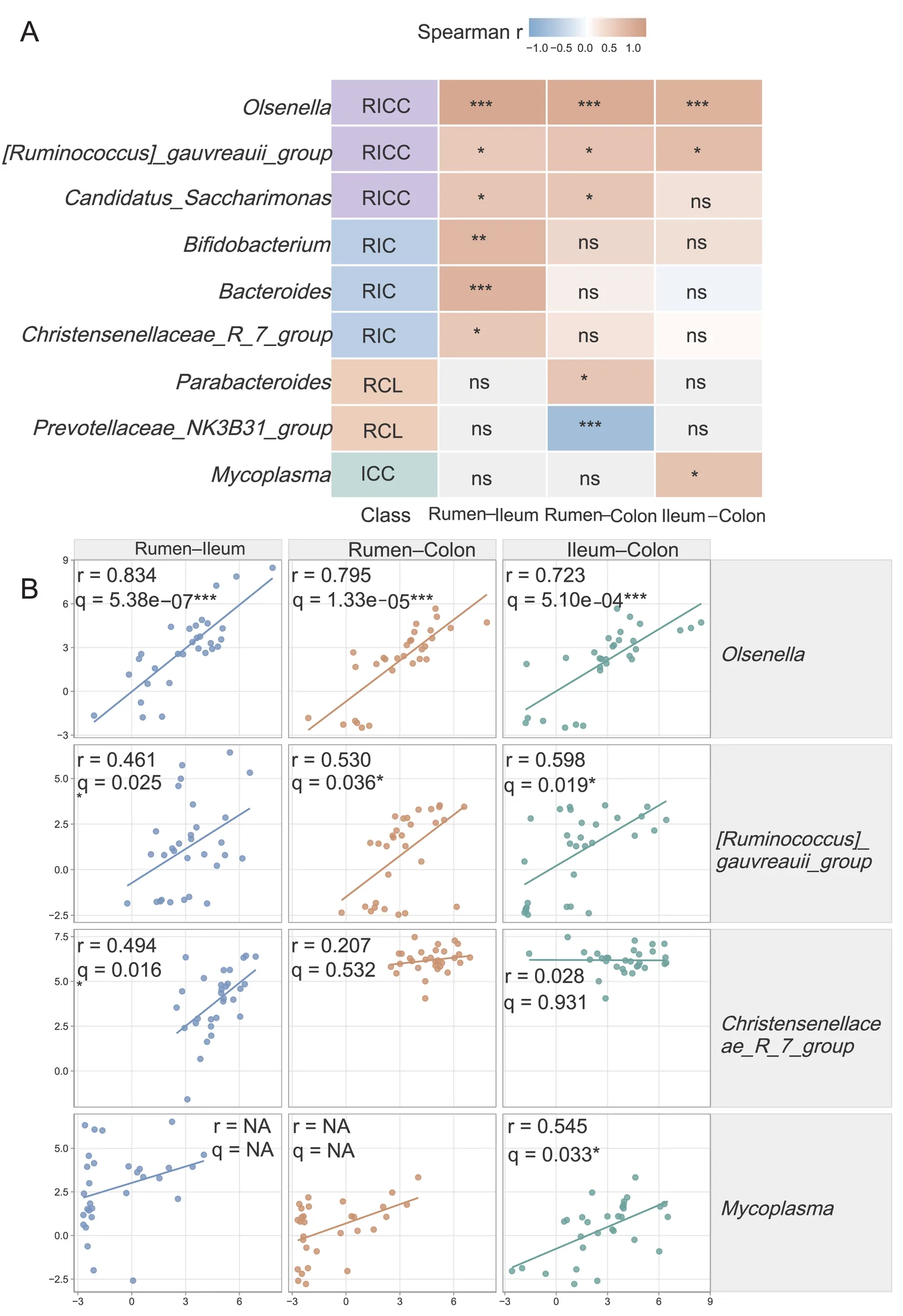
Structural continuity analysis identifies representative cross-compartment coordinated genera. (A) Correlation matrix for representative genera assigned to four structural continuity classes, including rumen–ileum–colon continuous genera (RICC), rumen–ileum continuous genera (RIC), rumen–colon linked genera (RCL), and ileum–colon continuous genera (ICC). (B) Scatter plots showing abundance relationships of representative genera across compartment pairs. Asterisks indicate statistical significance: *q < 0.05, **q < 0.01, and ***q < 0.001.

Among all genera included in the analysis, 66 showed evidence of cross-compartment continuity. Of these, genera showing continuity in the rumen–ileum comparison represented the largest group (51 genera), whereas only nine and six genera showed continuity in the rumen–colon and ileum–colon comparisons, respectively (Fig. 3A). When the three pairwise relationships were jointly considered for structural classification, rumen-ileum continuous genera represented the largest category (45 genera), whereas rumen-colon linked genera and ileum-colon continuous genera accounted for four and three genera, respectively. In addition, two genera showed strong three-compartment continuity (Fig. 3A). Strong three-compartment continuity referred to genera showing significant positive correlations in all three pairwise comparisons—rumen–ileum, rumen–colon, and ileum–colon.

Representative genera further illustrated this organization (Fig. 3B). *Olsenella* showed significant positive correlations in all three pairwise comparisons and therefore represented a typical full-length continuity pattern. *[Ruminococcus]_gauvreauii_group* showed a similar profile, indicating that whole-tract continuity was supported by more than one key genus. In contrast, *Christensenellaceae_R_7_group* was significant only in the rumen–ileum comparison, representing a more localized anterior continuity pattern. *Mycoplasma* mainly reflected hindgut continuity through its ileum–colon association.

### Pairwise correlation analysis reveals segment-specific associations with hepatic transcription and host metabolic phenotypes

**Figure 4:**
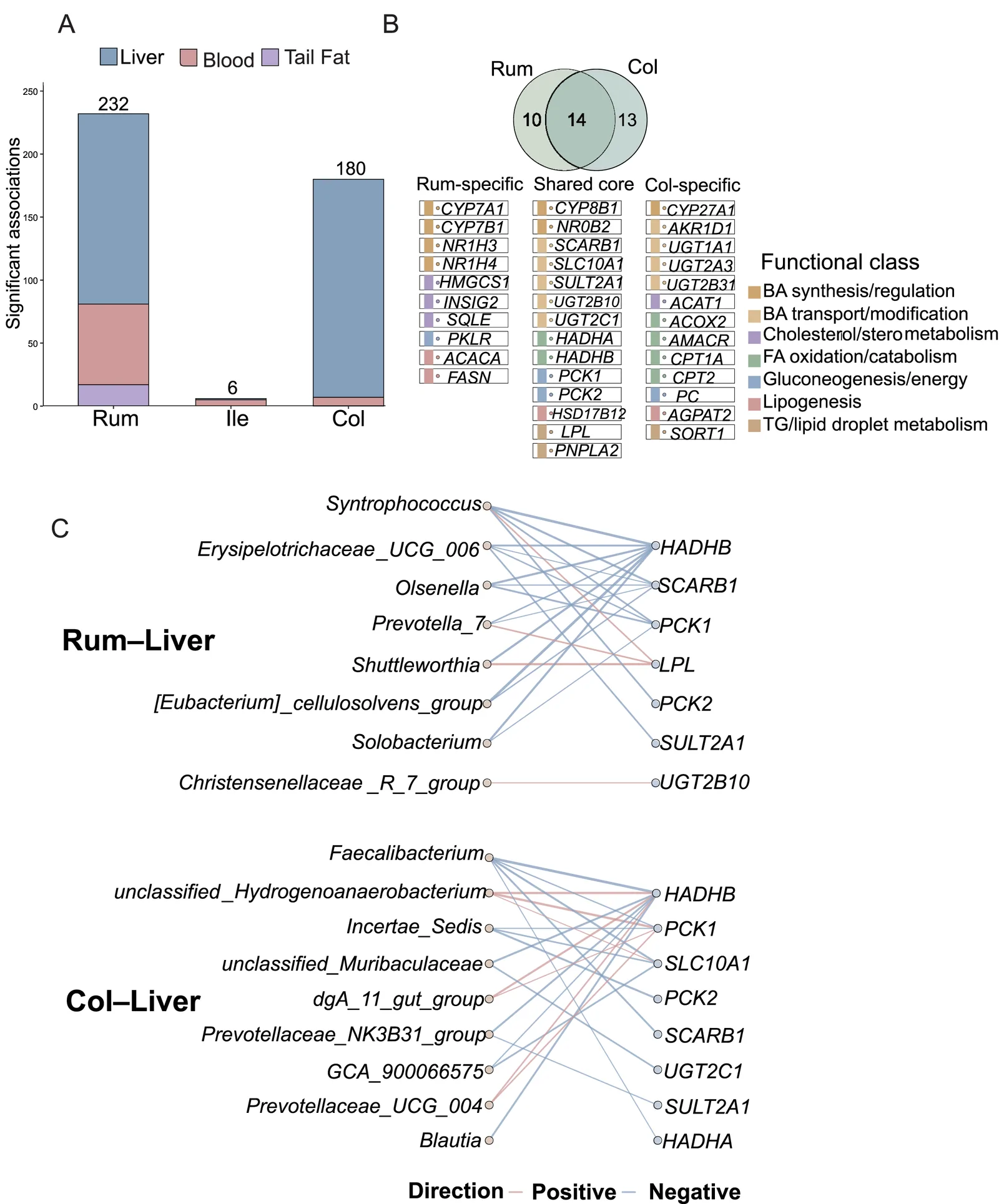
Pairwise associations reveal compartment-specific liver-linked microbial connection spectra. (A) Numbers of significant associations between compartment-specific microbiota and host layers. (B) Shared and compartment-specific liver genes associated with ruminal and colonic microbiota, with functional annotations. (C) Representative pairwise association networks linking ruminal or colonic genera to liver genes.

Pairwise correlation analysis revealed a compartment-partitioned association landscape with clear segment-specific features (Fig. 4A). When the hepatic genes associated with these two compartments were compared directly, rumen–liver and colon–liver associations converged on a common core set of genes while also retaining segment-biased features (Fig. 4B). The shared core genes included *SCARB1*, *SLC10A1*, *SULT2A1*, *UGT2C1*, *HADHA*, *HADHB*, *PCK1*, *PCK2*, and *HSD17B12*, suggesting that bile acid transport and metabolism, lipid oxidation, and gluconeogenesis represent recurrent association nodes shared by the rumen- and colon-related networks. At the same time, compartment-specific biases were also evident. The rumen was more often linked to *CYP7A1*, *NR1H3*, *SQLE*, *ACACA*, and *FASN*, whereas the colon was more often linked to *CYP27A1*, *AKR1D1*, *UGT1A1*, *ACOX2*, *CPT1A*, and *CPT2*.

Representative subnetworks supported this interpretation (Fig. 4C). In both rumen–liver and colon–liver networks, multiple microbial genera were statistically associated with a limited number of recurrent hepatic genes. In the rumen–liver network, genera such as *Olsenella*, *Prevotella_7*, *Shuttleworthia*, *[Eubacterium]_cellulosolvens*_*group*, and *Solobacterium* were linked to genes including *HADHB*, *SCARB1*, *PCK1*, *PCK2*, *SULT2A1*, and *UGT2B10*. In the colon–liver network, *Faecalibacterium*, *dgA-11_gut_group*, *Blautia*, *Prevotellaceae*_*UCG*-*004*, and *Prevotellaceae*_*NK3B31*_*group* were more frequently connected to *HADHB*, *PCK1*, *PCK2*, *SCARB1*, *SLC10A1*, *UGT2C1*, and *SULT2A1*. Within the blood trait-related modules, the rumen showed substantially more significant associations than either the ileum or colon. Significant tail fat-related associations were detected only in the rumen.

### sGCCA identifies trait-associated rumen-ileum-colon–liver association structures

**Figure 5:**
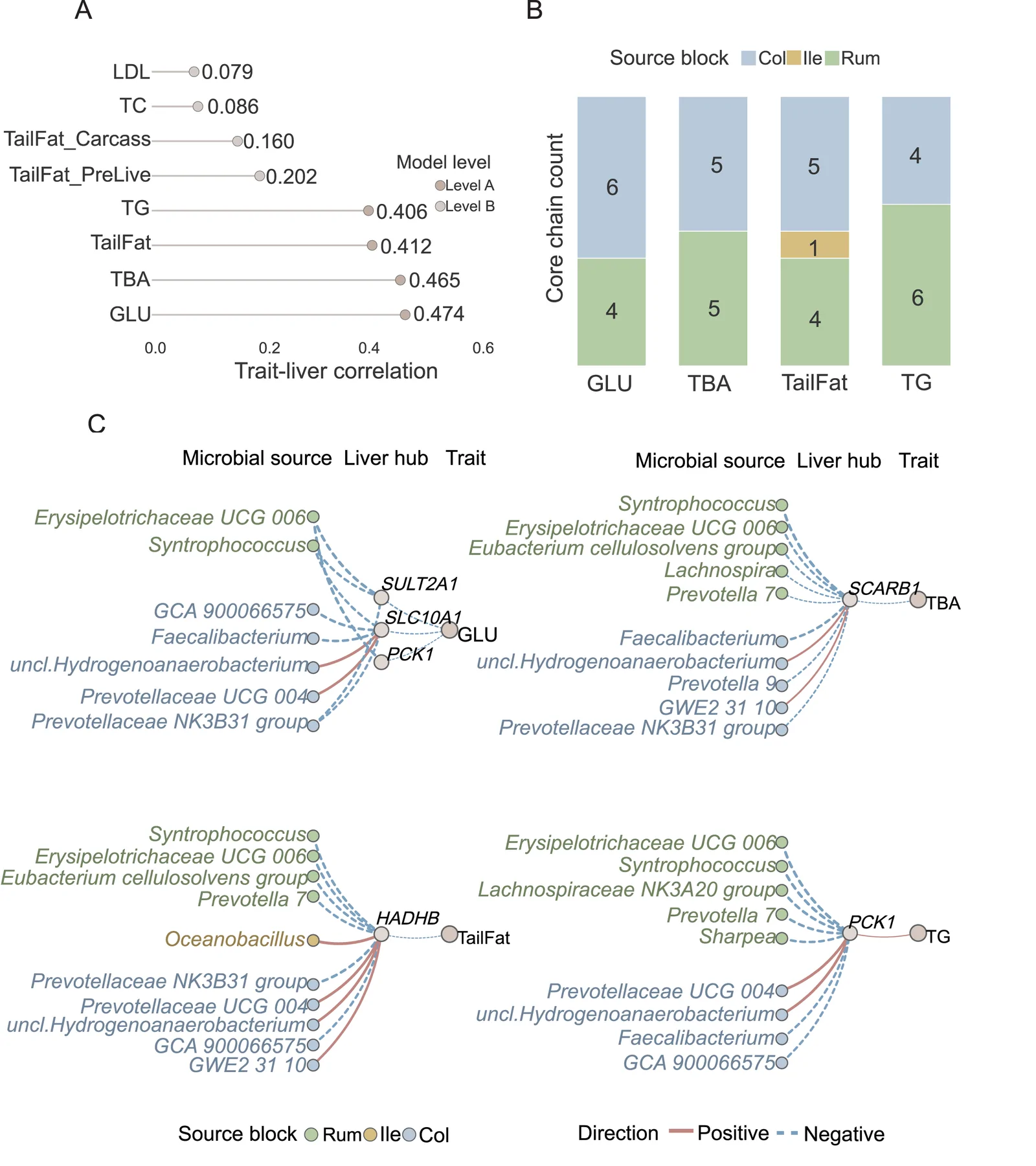
sGCCA identifies trait-related cross-omics association structures. (A) Trait–liver correlation strengths for each host trait. (B) Numbers of core chains for each trait by microbial source block. (C) Representative sGCCA-derived association networks involving microbial features, liver hub genes, and host traits. For readability, unclassified taxa are abbreviated as “uncl.” followed by the lowest assigned taxonomic name.

Among all traits examined, TBA, GLU, TG, and TailFat showed the strongest cross-omics convergence, whereas LDL, TC, and the two tail fat ratio traits showed substantially weaker integration (Fig. 5A). The TBA-associated structure showed the strongest cross-omics integration in this study. This structure was most prominently associated with the hepatic gene *SCARB1* and included microbial features from both the rumen and colon, whereas fewer ileal features were retained (Figs. 5B, 5C). Representative rumen genera within this core structure included *Syntrophococcus*, *Erysipelotrichaceae_UCG_006*, [*Eubacterium*]_*cellulosolvens*_*group*, *Lachnospira*, and *Prevotella_7*. The main colonic contributors included *Faecalibacterium*, unclassified *Hydrogenoanaerobacterium*, *Prevotella_9*, *GWE2_31_10*, and *Prevotellaceae_NK3B31_group*. Overall, this TBA-associated structure showed the clearest pattern of joint ruminal and colonic associations with a recurrent hepatic node related to bile acid homeostasis. The TG-associated structure was most prominently associated with *PCK1* and retained more ruminal than ileal or colonic microbial features. Representative rumen genera retained in its core structure included *Erysipelotrichaceae_UCG_006*, *Syntrophococcus*, *Prevotella_7*, and *Sharpea*, whereas the main colonic contributors included *Prevotellaceae_UCG_004*, *Faecalibacterium*, and *GCA 900066575*. By contrast, the GLU-associated structure retained relatively more colonic features, with *SLC10A1*, *SULT2A1*, and *PCK1* representing the principal hepatic nodes. Within this axis, the representative rumen genera were mainly *Syntrophococcus* and *Erysipelotrichaceae_UCG_006*, whereas the representative colonic genera included *GCA 900066575*, *Faecalibacterium*, *Prevotellaceae_UCG_004*, and *Prevotellaceae_NK3B31_group*. The TailFat-associated structure was most prominently associated with *HADHB* and, like the TBA-associated structure, included microbial features from both the rumen and colon. The main rumen-derived genera included *Syntrophococcus*, *Erysipelotrichaceae_UCG_006*, *[Eubacterium]_cellulosolvens_group*, and *Prevotella_7*. Only *Oceanobacillus* was retained from the ileum, whereas the colonic contributors included *Prevotellaceae_NK3B31_group*, *Prevotellaceae_UCG_004*, unclassified *Hydrogenoanaerobacterium*, *GCA_900066575*, and *GWE2_31_10*.

### Mediation analysis identifies candidate gut–liver–trait intermediate associations

**Figure 6:**
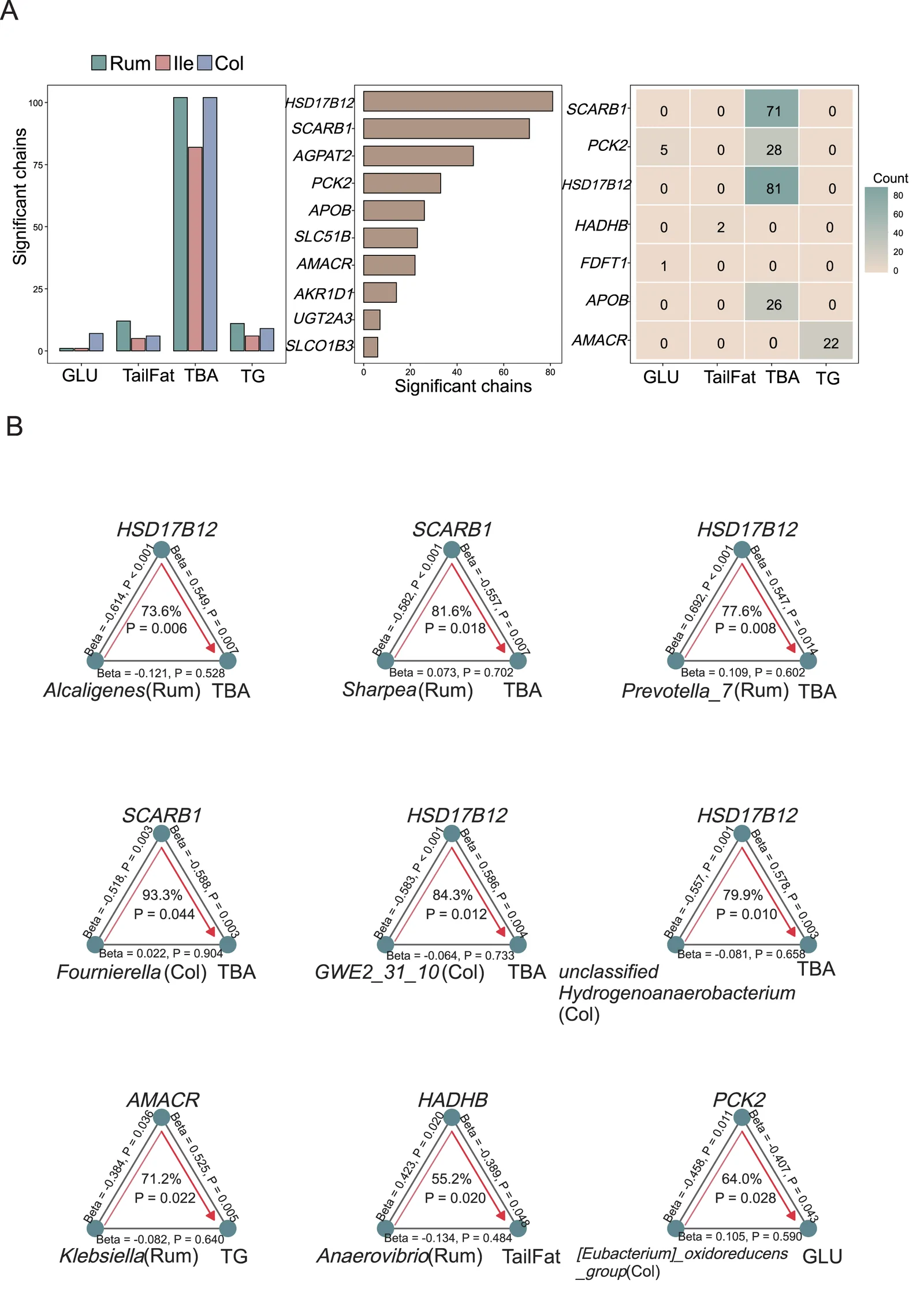
Mediation analysis identifies candidate intermediate associations involving hepatic genes in microbiota–host relationships. (A) Overall distribution of statistically significant candidate mediation chains, including trait-specific counts by microbial compartment, recurrent hepatic candidate mediators, and their distribution across host traits. (B) Representative statistically significant candidate mediation chains involving microbial genera, hepatic genes, and host traits.

Statistically significant candidate mediation chains were strongly concentrated in the serum TBA phenotype. Of the 344 significant chains identified, 286 were linked to TBA, whereas only 26, 23, and nine were linked to TG, TailFat, and GLU, respectively (Fig. 6A). The contributions of the three gut segments were not equivalent. The rumen, colon, and ileum contributed 126, 124, and 94 significant chains, respectively. At the mediator level, the significant chains were concentrated not across many hepatic genes, but around a limited set of recurrent transcriptional nodes, including *HSD17B12*, *SCARB1*, *PCK2*, *AMACR*, *HADHB*, and *APOB* (Fig. 6A). Among them, *HSD17B12* and *SCARB1* were predominantly involved in TBA-related chains. Colon-derived chains were more concentrated in the TBA module centered in *HSD17B12* and *SCARB1*, whereas rumen-derived chains, although also contributing to TBA, extended more broadly to TG-, GLU-, and TailFat-related phenotypes. Ruminal *Alcaligenes* and *Prevotella_7* were both classified as foregut-enriched genera and were included in candidate TBA-related mediation chains involving *HSD17B12* or *SCARB1*. In contrast, colon-derived *Fournierella* and *GWE2_31_10* were hindgut-enriched genera that showed statistically supported candidate intermediate associations with host phenotypes involving hepatic bile acid-related genes (Fig. 6B).

## Discussion

This study was designed to define the spatial organization of the ruminal, ileal, and colonic microbiota and provided a more complete view of how microbial variation across upstream and downstream gastrointestinal compartments is associated with bile acid-related gut–liver features in sheep. A central finding of this study is that microbial variation along the rumen–ileum–colon axis followed a directional and highly structured pattern. Previous work has shown that the ruminant gastrointestinal microbiota is spatially heterogeneous, with clear differences among anatomical segments in community composition, substrate use, and functional specialization (Zhang et al., 2025). Within this system, the rumen is specialized for the primary fermentation of complex plant substrates (Zhang et al., 2024a), the small intestine is more closely linked to nutrient absorption, transport, and host signaling (Zhang et al., 2024b), and the colon serves as a major site of distal anaerobic fermentation and microbial transformation of residual substrates and host-derived compounds (Fu et al., 2025; Yang et al., 2025). In addition to these anatomical and functional differences, evidence from dairy calves indicates that early microbial exposure can promote the colonization and persistence of specific bacterial taxa and facilitate the development of a stable hindgut microbiome, suggesting that the functional organization of the distal gut is shaped not only by local environmental conditions but also by microbial establishment and host-associated selection (Qiu et al., 2025). In the present study, the three compartments formed a hierarchical spatial continuum and showed distinct predicted functional profiles, suggesting that local ecological selection may shape both microbial taxonomy and inferred metabolic potential across the gastrointestinal tract.

The biological meaning of this spatial structure is illustrated by representative taxa. *Prevotella_7* and *Butyrivibrio* were enriched in the rumen and declined progressively towards the ileum, consistent with a strong preference for the fermentative environment of the foregut. This interpretation is supported by previous studies showing that *Prevotella* is a dominant ruminal microbiota involved in the degradation of cellulose, hemicellulose, and pectin, while also contributing to propionate production (Aschalew et al., 2025). *Butyrivibrio* is likewise a classical ruminal functional genus with established roles in fiber degradation and lipid biohydrogenation, linking it closely to both fermentation and lipid metabolism (Park, Seo & Kim, 2025). The distribution of these taxa therefore supports the view that the rumen acts as the major upstream fermentation niche in ruminants.

In contrast, *Anaerovibrio* and *Ruminococcus* showed a foregut–hindgut coordinated pattern, with relatively high abundance in both the rumen and colon and reduced abundance in the ileum. This suggests that the proximal and distal compartments share part of the ecological demand associated with anaerobic substrate fermentation and energy release. Previous studies have shown that *Anaerovibrio* participates in lipid decomposition in the rumen and contributes to the production of succinate and volatile fatty acids (Xie et al., 2025), whereas *Ruminococcus* is a core fibrolytic genus involved in the degradation of cellulose, pectin, and xylan (Xu et al., 2025a). Their spatial distribution in the present study therefore points to partial functional correspondence between the rumen and colon as the two major fermentative niches of the gastrointestinal tract.

Colon-enriched and gradient-changing taxa further refined this spatial framework. *Fournierella* and *Treponema* increased progressively from the rumen to the colon, supporting the view that the colon is a distinct distal fermentation niche. Although functional evidence for *Fournierella* in sheep remains limited, it has been described as a strictly anaerobic intestinal taxon that may be associated with short-chain fatty acid-related metabolism. By contrast, the role of *Treponema* is better established, and previous studies have shown that it is associated with pectin degradation and may be enriched under pectin-rich conditions (Liu et al., 2015). At the same time, *Romboutsia* and *Succiniclasticum* exhibited gradient-like redistribution across the tract. *Romboutsia* is typically regarded as adapted to nutrient-rich intestinal environments and capable of utilizing a broad range of carbohydrates (Wang et al., 2025b), whereas *Succiniclasticum* is well known for converting succinate to propionate (Ma et al., 2025). These taxa therefore reflect directional functional reorganization along the entire gastrointestinal tract.

Beyond spatial specificity, continuity analysis identified a second key feature of the ruminant gastrointestinal microbiota. Only a subset of genera showed clear cross-compartment continuity, which indicates that internal linkage across the gastrointestinal tract is selective and hierarchical rather than universal. *Olsenella* and [*Ruminococcus*]_*gauvreauii*_*group* showed positive correlations across all three pairwise comparisons, suggesting that full-length continuity is supported by a limited number of key taxa. By contrast, *Christensenellaceae_R_7_group* was more characteristic of upstream continuity, whereas *Mycoplasma* reflected downstream continuity. Available studies indicate that *Olsenella* is associated with the utilization of fermentable carbohydrates and the production of lactate and succinate (McLoughlin et al., 2020). *Christensenellaceae_R-7_group* has repeatedly been linked to volatile fatty acid production, rumen development, nutrient digestion and absorption, and fiber utilization in ruminants (Wang et al., 2025a). These observations suggest that cross-compartment continuity in the present study was not simply a pattern of synchronous fluctuation but may instead identify candidate gastrointestinal taxa whose coordinated abundance patterns are associated with ruminant metabolism.

At the host interaction level, pairwise correlation analysis showed that the colon formed abundant associations with hepatic transcription and, together with the rumen, represented one of the two gastrointestinal compartments showing the broadest statistical associations with the liver transcriptome. Colon microbial fermentation is a major hepatic coupling interface (Yang et al., 2025). The current study also found that the rumen displayed a broader range of statistical associations with blood biochemical traits and tail fat phenotypes, and significant tail fat-related associations were detected only in the rumen module. Rumen-associated taxa, including *Prevotella_7*, *Olsenella*, and other fermentation-related microbes, are compatible with an upstream niche specialized in substrate degradation and fermentation. By contrast, colon-associated taxa are involved in anaerobic fermentation such as *Faecalibacterium*, *Prevotellaceae_UCG-004*, and *Prevotellaceae_NK3B31_group*. *Faecalibacterium* is widely recognized as a butyrate-producing genus (Khan et al., 2025), which fits well with its appearance in the colon–liver network. These differences support a complementary model in which the colon is linked more closely to distal fermentation, whereas the rumen provides the major upstream fermentation.

The sGCCA analysis further showed that these associations were not isolated pairwise correlations but were organized into coherent cross-omics structures. The strongest integrative structures were found in rumen and colon microbiota that were associated with TBA, TG, GLU, and TailFat, with the TBA-associated structure showing the strongest cross-omics coherence. Notably, this structure was prominently associated with *SCARB1*, which is consistent with its role in cholesterol uptake and its relevance to bile acid homeostasis (Ma et al., 2020). The TG- and TailFat-related structures were prominently associated with *PCK1* and *HADHB*, respectively, linking microbial variation to hepatic pathways involved in gluconeogenesis (Zhang, Koser & Donkin, 2021) and mitochondrial fatty acid β-oxidation (Xu et al., 2025b). These results suggest that ruminal and colonic microbial variation is statistically integrated with recurrent hepatic metabolic features, rather than being restricted to compartment-level microbial variation.

Mediation analysis evaluated candidate directional relationships within these associations under the specified statistical models. Significant mediation chains were concentrated on *HSD17B12*, *SCARB1*, *PCK2*, *AMACR*, and *HADHB*. *HSD17B12* has been implicated in adipogenic differentiation and lipid accumulation in sheep (Liu et al., 2023), *PCK2* is a recognized regulator of hepatic gluconeogenesis (Yang et al., 2024), *HADHB* is linked to mitochondrial fatty acid β-oxidation (Xu et al., 2025b), *SCARB1* encodes the HDL receptor SR-BI and participates in hepatic cholesterol handling within bile acid-related homeostatic programmes (Ma et al., 2020), and *AMACR* is involved in the metabolism of branched-chain fatty acids and bile acid intermediates, although direct evidence in sheep remains limited. Thus, these mediation patterns provide statistical support for their potential involvement in associations related to bile acid homeostasis, glucose metabolism, and lipid metabolism. Representative mediation chains in the present study identified a candidate statistical chain involving the rumen-enriched genus *Prevotella_7*, hepatic *SCARB1*, and serum TBA. Previous studies have shown that *Prevotella* may participate in bile acid- and FXR-related regulation (Jiang et al., 2022), and that *SCARB1/SR-BI* regulates hepatic cholesterol uptake, thereby linking this gene to bile acid precursor availability (Malerød et al., 2005). In addition, mediation analysis identified a candidate statistical chain involving the continuity-associated genus *Anaerovibrio*, hepatic *HADHB*, and TailFat. Previous studies have shown that *Anaerovibrio lipolytica* is a typical ruminal lipolytic bacterium involved in ruminal lipid hydrolysis (Prins et al., 1975), whereas *HADHB* encodes a subunit of the mitochondrial trifunctional protein and participates in long-chain fatty acid β-oxidation (Xu et al., 2025b). This association pattern extends the potential relevance of the rumen beyond local fermentation and supports its consideration as a candidate upstream microbial component in studies of the ruminant gut–liver axis.

Overall, our findings support consideration of an expanded conceptual model of the ruminant gut–liver axis in which the rumen, ileum, and colon form a spatially structured system with coordinated microbial and host-associated patterns. These findings support the use of a rumen–intestine–liver analytical framework in ruminants rather than a strictly intestine-centered gut–liver model. Because this study was observational and cross-sectional, all correlation analyses, sGCCA, and mediation analyses should be interpreted as statistical associations or candidate intermediate relationships rather than evidence of direct regulation or causality. A further limitation is that the bile acid-related phenotype examined in this study was restricted to serum TBA concentrations together with hepatic transcriptional changes in bile acid metabolism- and signaling-related genes. Therefore, our findings primarily reflect associations with a systemic bile acid-related phenotype and hepatic transcriptional regulation, rather than a comprehensive characterization of bile acid synthesis, microbial biotransformation, enterohepatic circulation, or receptor-mediated signaling. In addition, microbial functional profiles were inferred from 16S rRNA sequencing data using PICRUSt2 and therefore represent predicted functional potential rather than directly measured microbial activity. Differences in predicted KO and pathway abundance do not demonstrate microbial gene expression or the occurrence of corresponding enzymatic reactions *in vivo*. Future studies integrating functional validation approaches, including metagenomics, metatranscriptomics, metaproteomics, targeted bile acid profiling, enzyme activity assays, and longitudinal or intervention-based designs, will be required to further determine the mechanistic roles of identified microbial taxa and hepatic transcriptional changes in bile acid-related metabolic regulation. These findings should be interpreted within the context of Tan lambs maintained under the same experimental conditions and may not be directly generalizable to other breeds, ages, diets, or production systems without further validation. Nevertheless, the paired sampling design across rumen, ileum, and colon provides a useful framework for evaluating compartment-specific and coordinated microbial contributions to host metabolism in ruminants.

## Conclusion

Our study shows that the rumen, ileum, and colon in sheep are spatially distinct yet structurally connected microbial compartments. By integrating microbiota, hepatic transcriptomic features, and host phenotypes, we found that compartment-specific microbial variation showed recurrent statistical associations with a limited set of hepatic metabolic genes, particularly *SCARB1*, *HSD17B12*, *PCK1*/*PCK2*, and *HADHB*, with serum TBA-related associations representing the dominant host-associated pattern. Together, these findings support consideration of the ruminant gut–liver axis as a multi-compartment framework that includes potential upstream ruminal associations, distal colonic microbial patterns, and related hepatic transcriptional features. Within this framework, the rumen emerges not only as a fermentation chamber, but also as a candidate upstream microbial component associated with host metabolic variation.

## Supplemental Information

10.7717/peerj.21705/supp-1 Supplemental Information 1 ARRIVE 2.0 Checklist.

10.7717/peerj.21705/supp-2 Supplemental Information 2 Hepatic candidate genes involved in bile acid and glycolipid metabolism.

10.7717/peerj.21705/supp-3 Supplemental Information 3 Ingredients and nutrient composition of the basal diet.

10.7717/peerj.21705/supp-4 Supplemental Information 4 Computer code.

10.7717/peerj.21705/supp-5 Supplemental Information 5 Rumen and colon-centered guy-liver axis in sheep.

## Additional Information and Declarations

### Data Availability

The following information was supplied regarding data availability:

The code is available in the Supplemental Files.

The code is available at GitHub and Zenodo:

- https://github.com/gaoyuyang741-lab/sheep-gut-liver-microbiota-code/tree/v1.0.1

- gaoyuyang741-lab. (2026). gaoyuyang741-lab/sheep-gut-liver-microbiota-code: v1.0.1 English code comments (Version v1.0.1) [Computer software]. Zenodo. https://doi.org/10.5281/zenodo.20543366

The raw sequence data are available at NCBI: PRJNA1158691, PRJNA1156902, PRJNA1156904, PRJNA1158020.
