# Supplementary material for: Rumen and colon-centered microbiota-liver interactions associated with bile acid-related metabolic regulation in sheep
Source: PeerJ. 2026 Sep 10;14:e21705. doi: 10.7717/peerj.21705 (PMC13571605; doi:10.7717/peerj.21705)
Supplement: Supplementary file 2 [file peerj-14-21705-s005.pdf]

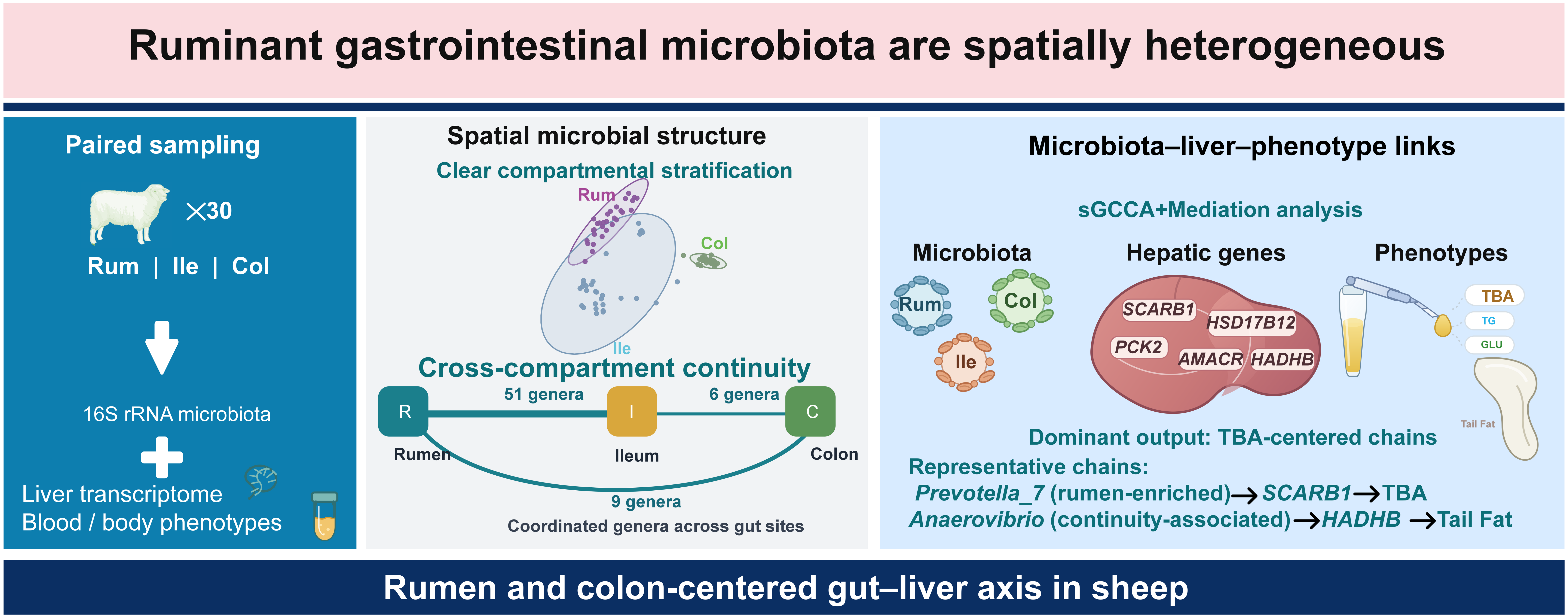
Ruminant gastrointestinal microbiota are spatially heterogeneous
Paired sampling
×30
Rum | Ile | Col
16S rRNA microbiota
Liver transcriptome
Blood / body phenotypes
Spatial microbial structure
Clear compartmental stratification
Rum
Col
Ile
Cross-compartment continuity
51 genera
6 genera
R
I
C
Rumen
Ileum
Colon
9 genera
Coordinated genera across gut sites
Microbiota–liver–phenotype links
sGCCA+Mediation analysis
Microbiota
Hepatic genes
Phenotypes
Rum
Col
Ile
SCARB1
HSD17B12
PCK2
AMACR
HADHB
TBA
TG
GLU
Tail Fat
Dominant output: TBA-centered chains
Representative chains:
Prevotella_7 (rumen-enriched)→SCARB1→TBA
Anaerovibrio (continuity-associated)→HADHB →Tail Fat
Rumen and colon-centered gut–liver axis in sheep
